# Supplementary material for: An appraisal of fund of funds efficiency based on risk-adjusted performance measures: Application of an augmented WASPAS methodology
Source: PLoS One. 2025 Jul 23;20(7):e0314918. doi: 10.1371/journal.pone.0314918 (PMC12286336; doi:10.1371/journal.pone.0314918)
Supplement: Attachment — Submitted filename: Response to Reviewers.docx [file pone.0314918.s003.docx]

***COVER LETTER FOR*** ***RESUBMISSION OF MANUSCRIPT***

Dear Academic Editor Gabrijela Popovic.

We have submitted a thoroughly revised version of our manuscript, titled *"An Appraisal of Fund of Funds Efficiency Based on Risk-Adjusted Performance Measures: Application of an Augmented WASPAS Methodology,"* for your consideration for publication in *PLOS ONE.*

We extend our profound gratitude to the reviewers for their incisive and invaluable feedback, which has played a pivotal role in enhancing the rigor and quality of our manuscript. Accompanying this letter are meticulously crafted responses to each of the reviewers' comments. Additionally, we have implemented comprehensive revisions aimed at elevating both clarity and precision. These modifications encompass refinements to the textual narrative and an enriched literature review, effectively addressing areas that previously exhibited ambiguity or lacked completeness.

We eagerly await your response at your earliest convenience.

Sincerely yours,

***Rouzbeh Ghousi****, Associate Professor*

***Title of the paper:*** *An Appraisal of Fund of Funds Efficiency Based on Risk-Adjusted Performance Measures: Application of an Augmented WASPAS Methodology*

The authors would like to extend their sincere gratitude to all the reviewers and the Associate Editor for their dedicated efforts and valuable time spent reviewing and enhancing our paper. In light of their constructive feedback and insightful recommendations, we have meticulously revised the manuscript in alignment with the referees' comments.

Below, we present our responses to each of the referees' comments. All revisions and changes in the updated manuscript have been highlighted in yellow for clarity.

**Responses to the comments of the reviewers and editors**

| **Reviewer 1** | |
| --- | --- |
| (1) | ***The article is well written in standard American English, though a few minor issues could be addressed for enhanced clarity. The existing literature could be better cited because the article lacks the most recent respectable articles. For example, there are no papers from 2024, only two from 2023, only one from 2022, no references from 2021 and only two references from 2020. Altogether, the article has only five references from the last five years. Authors should consult more recent literature to make their research up to date.*** |
| Response | Dear reviewer,  We extend our sincere gratitude for your meticulous review and constructive comments. Your observation regarding the need to integrate more recent literature to enhance the manuscript’s contemporaneity and scholarly foundation is well-taken. In response, we have undertaken a comprehensive update of our references, incorporating a significant number of recent publications from 2021 to 2024, thereby ensuring a more thorough representation of the latest advancements in the field. These additions, highlighted in the revised manuscript, strengthen the contextual framework and underscore our commitment to aligning with the current discourse. We deeply appreciate your insights, which have been instrumental in elevating the academic rigor and clarity of our work. |
| Action | Yuan and Yuan [14] propose a comprehensive multi-index ranking method for mutual fund performance, addressing the limitations of existing ranking approaches. Traditional single-index methods are often inadequate, while multi-index methods tend to be indirect and inflexible. The new method combines both cardinal and ordinal data, offering a more accurate and robust ranking. It also introduces a novel objective weighting approach to address index correlation issues. This flexible and practical method provides a clearer and more reliable evaluation of mutual fund performance, making it a valuable tool for investors and researchers.  Malhotra et al. [15] offer a detailed analysis of the performance of financial mutual funds, focusing on a 23-year period significantly longer than previous studies. The research incorporates multiple performance evaluation techniques, such as risk-adjusted returns, multifactor analysis, and market timing, providing a holistic view of fund behavior. The findings indicate that financial mutual funds outperform both the market and sector-specific benchmarks in terms of risk-adjusted returns, though their alphas remain indistinguishable from zero. Moreover, the study reveals that fund managers do not possess market timing or security selection skills. The authors further dissect performance trends across different periods, including the pre- and post-2008 financial crisis and the recent COVID-19 pandemic, offering insights into the stability and adaptability of financial mutual funds under various market conditions.Top of FormBottom of Form  Mota et al. [16] introduce a new hybrid multiple-criteria decision-making (MCDM) methodology, BWM-Moora-N, for prioritizing investment funds. The study combines the Best-Worst Method (BWM) with the Multi-Objective Optimization by Ratio Analysis (MOORA) to address the complexities inherent in evaluating and selecting investment funds. This method overcomes the limitations of traditional MCDM approaches by improving accuracy in the prioritization process, particularly in scenarios involving a large number of criteria. The authors demonstrate the effectiveness of the proposed model through empirical analysis, showing how it provides a more structured and reliable decision-making framework for investors, thereby enhancing the strategic selection of investment funds in diverse market conditions.  [14] J. Yuan and X. Yuan, “A Comprehensive Method for Ranking Mutual Fund Performance,” Sage Open, vol. 13, no. 2, Apr. 2023, doi: 10.1177/21582440231174176.  [15] D. K. Malhotra, T. Mooney, R. Poteau, and P. Russel, “Assessing the Performance and Risk-Adjusted Returns of Financial Mutual Funds,” International Journal of Financial Studies, vol. 11, no. 4, p. 136, Nov. 2023, doi: 10.3390/ijfs11040136.  [16] V. R. R. Mota, C. F. S. Gomes, D. F. de Lima Silva, and M. dos Santos, “The novel hybrid multiple criteria decision method BWM-Moora-N applied for investment funds prioritization,” Operational Research, vol. 24, no. 3, p. 36, Sep. 2024, doi: 10.1007/s12351-024-00817-w. |
| (2) | ***The abstract is written clearly without references and has 226 words, less than the maximum length. The abstract describes the main objective of the research and explains the methodology without too many details. According to the journal's instructions, the abbreviations should not be included if possible. The recommendation for authors is to modify the abstract and retain fewer abbreviations. It is clear that some of the abbreviations have to remain, but I firmly believe that authors can rewrite the abstract to include less than 12 abbreviations as they have in the abstract at the moment.*** |
| Response | Thank you for your insightful suggestion regarding the use of abbreviations in the abstract. We appreciate your attention to detail and your guidance on adhering to the journal’s requirements. |
| Action | In response, we have carefully revised the abstract to reduce the number of abbreviations to fewer than twelve, retaining only those essential for clarity and conciseness. This modification aligns with the journal's guidelines and enhances readability for a broader audience.  In the complex landscape of investment management, Fund of Funds play a crucial role in constructing diversified portfolios with professional oversight. However, evaluating the performance of Fund of Funds is a challenging task due to their intricate management layers and diverse underlying assets. Traditional performance assessment methodologies often fall short in capturing these complexities, leading to potential inefficiencies in decision-making. This study addresses these challenges by applying a Multi-Attribute Decision Making approach, specifically an augmented Weighted Aggregated Sum Product Assessment methodology, to provide a comprehensive appraisal of Fund of Fund performance. This study introduces an innovative modification to the Weighted Aggregated Sum Product Assessment methodology, addressing its inherent limitations and providing a more advanced evaluative framework. Despite its ability to offer a structured basis for decision-making, this approach has certain constraints, particularly in identifying the most influential performance criteria impacting outcomes. To resolve this issue, the proposed modification incorporates entropy measures and effective weighting techniques, thereby enhancing the robustness and precision of performance evaluation. This approach provides a sophisticated evaluative framework, facilitating investors in making informed decisions and optimizing investment strategies. This methodology was tested on a set of Iranian Fund of Funds, with computational results demonstrating the strong performance and robustness of the proposed approach. Among the alternatives evaluated, TMSK emerged as the best-performing fund, highlighting the effectiveness of the modified Weighted Aggregated Sum Product Assessment method in facilitating informed investment decisions and optimizing portfolio strategies. |
| (3) | ***The introduction is written like a funnel, providing broad information regarding the research issue and then narrowing it. However, the introduction is almost four pages long, and the authors should consider dividing it. It also lacks referencing methods and models in the text currently in the introduction. The methodology is conducted to a respectable technical standard and is documented adequately. However, the authors should elaborate more on their choice of the WASPAS method rather than some of the more recent methods. The conclusions drawn are clearly articulated and well-supported by the data presented.*** |
| Response | Thank you for your valuable feedback and constructive recommendations regarding the structure and content of the introduction and methodology sections. Your observations are greatly appreciated and have been instrumental in guiding our revisions. |
| Action | In response, we have improved the structure of the introduction through a careful revision to enhance readability and clarity, while maintaining it as a cohesive section without dividing it further. Additionally, we have enriched the introduction by incorporating relevant references to key methods and models, addressing the need for a well-supported contextual framework. In the methodology section, we have expanded our discussion on the rationale for selecting the WASPAS method, explaining its advantages over more recent approaches and its suitability for our research objectives. We trust these enhancements address your concerns and contribute to the overall rigor and coherence of the manuscript. |
| (4) | ***Several segments could be improved regarding the formatting of the paper. For example, Table 8 should be written in capital first letter. There needs to be an introduction of Figure 2 in the integral text. Numbers in Figure 3 are written in different fonts. Numbers 2 and 4 should be further away from the chart to be observed more clearly. The manuscript should be formatted by the instructions to meet the journal's formatting requirements.*** |
| Response | Thank you for your careful review and attention to the formatting details of the manuscript. We appreciate your suggestions, which are essential to ensuring that the paper adheres to the journal’s standards and presents the information clearly. |
| Action | In response, we have made the recommended formatting adjustments throughout the manuscript. Specifically, we have revised Table 8 to start with a capital letter, introduced Figure 2 within the text, and standardized the font in Figure 3. Additionally, we have adjusted the positioning of numbers 2 and 4 in Figure 3 to improve their clarity. These changes bring the manuscript in closer alignment with the journal's formatting guidelines and enhance the overall presentation of our work. |
| (5) | ***There are also specific issues in the reference list. Several references are missing certain segments. For example, Keating & Shadwick (2002), Treynor (1965), Treynor (1966), Young (1991) and several others need pagination; Fox (2016) needs to include the name of the book, etc. Authors are advised to check references and unify the list. Some references are written in all caps, some with capitalisations of titles and others regularly. All references have to be written according to the journal rules.*** |
| Response | Thank you for your thorough review and for highlighting the specific issues in the reference list. Your attention to these details is invaluable for ensuring the accuracy and consistency of our citations. |
| Action | In response, we have carefully reviewed and corrected the reference list to address the noted issues. Missing segments, such as pagination for references like Keating & Shadwick (2002), Treynor (1965, 1966), and Young (1991), have been added. We also included the book title for Fox (2016) and standardized the formatting of all references to align with the journal's guidelines, ensuring uniform capitalization throughout. These revisions enhance the precision and adherence of our manuscript to the required referencing standards.  [34] C. Keating and W. Shadwick, “A Universal Performance Measure,” Journal of Performance Measurement, vol. 6, pp. 59–84, Jan. 2002.  [17] W. P. Fox, “Multi-Attribute Decision Making in Risk Analysis,” in Analyzing Risk through Probabilistic Modeling in Operations Research, IGI Global, 2016, pp. 246–290. doi: 10.4018/978-1-4666-9458-3.ch011.  [35] J. L. Treynor and K. G. Mazuy, “Can mutual funds outguess the market?,” Harv Bus Rev, pp. 131–136, 1966. |
| **Reviewer 2** | |
| (1) | ***The studies employing the WASPAS approach are not elaborated upon comprehensively.*** |
| Response | Thank you for your observation regarding the discussion of studies employing the WASPAS approach. We appreciate your input, as it is essential to provide a thorough context for the chosen methodology. |
| Action | In response, we have expanded our discussion to include a more comprehensive review of studies that utilize the WASPAS approach. This added elaboration highlights the relevance, application, and benefits of WASPAS in similar research contexts, thereby strengthening the methodological foundation of our manuscript. We trust that this enhancement provides a more complete understanding of the WASPAS approach within our study.  Fox [17] characterizes MADM as a structured method for evaluating and ranking options across multiple criteria. Widely applied across various fields, this approach strengthens decision-making by thoroughly considering each pertinent attribute of the available choices. Among MADM techniques, the WASPAS framework is notable for its dual-weighted mechanism, which enables a more refined aggregation of criteria, facilitating precise prioritization and selection among alternatives.  Konstants [18] emphasizes that utilizing the MADM framework, especially through the advanced capabilities of the WASPAS method, equips investors and decision-makers with a structured approach for systematically assessing and comparing investment options. By synthesizing multiple financial indicators and risk parameters, this rigorous analysis facilitates the precise identification of optimal investment prospects, maximizes potential returns, and strengthens risk management strategies. |
| (2) | ***The benefits of the Augmented WASPAS approach compared to the WASPAS method are not thoroughly delineated.*** |
| Response | Thank you for highlighting the need for a clearer distinction between the benefits of the Augmented WASPAS approach and the traditional WASPAS method. We appreciate your suggestion, as it adds valuable depth to our methodological discussion. |
| Action | In response, we have expanded the methodology section to provide a more detailed explanation of the advantages of the Augmented WASPAS approach compared to the standard WASPAS method. This includes a discussion of the specific enhancements and improvements offered by the augmented version in terms of accuracy, flexibility, and applicability to our research objectives. We believe this clarification strengthens the methodological rigor and justification within the manuscript.  The augmented WASPAS approach significantly refines traditional WASPAS by incorporating entropy-based weighting, which provides a more objective and precise weighting of criteria, thus reducing bias and amplifying the impact of the most informative criteria on the decision-making process. This enhanced sensitivity to performance differentials across multiple attributes overcomes the limitations of the original method, enabling a more sophisticated evaluation of complex investment portfolios. |
|  | ***Examination of Table 10 reveals significant discrepancies in the rankings. The discrepancies require elucidation.*** |
| response | Thank you for your insightful observation regarding the discrepancies in the rankings presented in Table 10. We appreciate your attention to this detail, as it is crucial for readers to understand the underlying reasons for these variations. |
| action | In response, we have added a detailed explanation in the results section to clarify the significant discrepancies observed in the rankings in Table 10. This additional commentary provides insights into the factors contributing to these variations, offering a clearer understanding of the ranking methodology and the contextual differences influencing each result. We trust this explanation addresses your concerns and enhances the interpretability of our findings.  The observed discrepancies in rankings stem from the fundamental differences in each methodology’s mathematical structure and underlying evaluation principles. Each approach, including WASPAS, ARAS, MARCOS, EDAS, MOORA, COPRAS, MABAC, MACBETH, and TOPSIS, employs distinct weighting schemes and aggregation mechanisms that place varying degrees of emphasis on particular aspects of the decision criteria. Consequently, these unique methodological attributes lead to different prioritization and ranking outcomes across alternatives when comparing them with the augmented WASPAS approach. This variability underscores the need to carefully consider the choice of method, as it can critically shape the decision-making process and the conclusions drawn from it. In this paper, the WASPAS methodology was selected due to its robust multi-criteria decision-making framework, which effectively balances both additive and multiplicative aggregation approaches. This dual approach allows WASPAS to capture a comprehensive picture of alternative performances, making it particularly well-suited for evaluating complex investment structures such as Fund of Funds. However, to address certain limitations inherent in the traditional WASPAS model such as its sensitivity to weighting schemes and its limited ability to identify the most influential performance criteria an augmented version of WASPAS was developed. This enhancement incorporates entropy-based weighting and refined evaluative techniques, thereby improving its robustness, precision, and ability to provide a more nuanced assessment of Fund of Funds performance. |
